# Informal caregiving for people with dementia and hearing or vision impairment: A systematic review

**DOI:** 10.1002/alz.70525

**Published:** 2025-08-14

**Authors:** Shaoqing Ge, Katie Trainum, Yaolin Pei, Wonshik Chee, Yuting Song, Bo Xie

**Affiliations:** ^1^ School of Nursing, The University of Texas at Austin Austin Texas USA; ^2^ School of Nursing, Qingdao University Qingdao China; ^3^ Faculty of Nursing University of Alberta Edmonton Alberta Canada; ^4^ School of Information The University of Texas at Austin Austin Texas USA

**Keywords:** Alzheimer's disease and related dementias, caregiving, hearing impairment, sensory, vision impairment

## Abstract

Caregivers of individuals with dementia often face the dual challenge of managing dementia alongside sensory impairments (hearing and/or vision loss). Despite the significant burden, this issue remains underexplored, with existing research largely overlooking the impact of sensory impairments on caregivers’ experiences. This systematic review examines the challenges and needs of caregivers managing both conditions. A comprehensive literature search across three databases identified 12 studies published before May 2024 in five countries. The review revealed that caregivers face increased challenges when managing both dementia and sensory impairments. Unmet needs, particularly in accessing tailored support and assistive technologies, were prevalent. Multidisciplinary care and interventions are crucial to address both cognitive and sensory needs. To improve the quality of life for caregivers and sensory impaired dementia patients, comprehensive support systems, enhanced caregiver education, and better access to affordable assistive technologies are essential. Addressing these disparities is critical for providing effective care.

**Highlights:**

- This review focuses on informal caregiving for people with dementia and sensory loss.
- Caregivers face increased challenges due to managing both dementia and sensory impairments.
- There are additive implications of caring for people with dual impairments.
- Further research is needed on interventions to support this caregiver population.

## 1 INTRODUCTION

The intersection of dementia and age‐related sensory impairments presents a significant challenge for dementia caregivers, who often bear the brunt of the physical, emotional, and financial burdens associated with caregiving. Dementia is a progressive neurological disorder affecting ≈ 50 million people globally, a number projected to triple by 2050 as populations age.^1^ Concurrently, hearing loss and vision impairment affect > 1.5 billion and 2.2 billion people worldwide, respectively,^2^, ^3^ and the prevalence of both conditions increases dramatically with age.^3^, ^4^ The co‐occurrence of these conditions can exacerbate the difficulties faced by both individuals with these impairments and their caregivers, creating a unique caregiving dynamic that demands focused attention and support.

Informal caregivers of people with dementia and sensory impairments face multifaceted challenges. They must navigate the complexities of managing cognitive decline along with communication barriers due to hearing loss and difficulties with visual perception. These overlapping impairments can lead to increased caregiver burden, higher levels of stress, frustration, isolation, and diminished quality of life for both caregivers and care recipients.^5^, ^6^ Understanding the specific experiences and needs of these caregivers is crucial for developing targeted interventions and support systems that can alleviate their burden and improve care recipients’ quality of life.

Despite growing recognition of these challenges, there is a lack of comprehensive literature synthesizing the experiences of caregivers who manage the dual burdens of dementia and sensory impairments. Previous literature reviews on the experiences of informal caregivers of people with dementia have predominantly centered on caregiver burden, often overlooking the role of patients’ hearing and vision impairments.^7^, ^8^, ^9^ Similarly, studies that have specifically examined hearing or vision impairments in dementia patients have seldom explored the impact of these sensory impairments on caregivers themselves.^10^, ^11^ Therefore, in this systematic review, we aim to: (1) synthesize and evaluate existing research on the burden of caregivers for individuals with dementia and co‐occurring hearing and/or vision impairments; and (2) critically examine their unmet needs and assess potential strategies to support these caregivers. In doing so, we seek to provide a holistic understanding of the caregiving experience in this context, to identify critical areas for intervention, and suggest directions for future research.

## 2 METHODS

This review follows the Preferred Reporting Items for Systematic Reviews and Meta‐Analyses (PRISMA) 2020 protocol for systematic literature reviews^12^; a PRISMA checklist is provided in Appendix S1 in supporting information. A protocol for the review is registered with PROSPERO.^13^

### 2.1 Search strategy

On May 8, 2024, we searched by titles and abstracts in the PubMed, CINAHL Plus with Full Text, and PsycINFO databases, using the following search terms: (dement* OR Alzheimer* OR ADRD OR mild cognitive impairment*) AND ((visually impaired OR visual impairment* OR vision disorder* OR blindness OR vision loss OR visual loss OR vision acuit* OR visual acuit* OR partial sight) OR (hearing loss OR hearing impairment* OR hearing deficit* OR hearing dysfunction* OR hearing disorder* OR hard of hearing OR impaired hearing OR loss of hearing OR auditory impairment* OR auditory deficit* OR auditory dysfunction* OR auditory acuit* OR deaf*)) AND (caregiv*). In PubMed, we included the following Medical Subject Headings as additional search terms: “dementia,” “visually impaired persons,” “vision disorders,” “hearing loss,” “persons with hearing impairments,” and “caregivers.” We imposed no limit on years of publication. The search terms were informed by the authors’ previous experience and existing literature reviews in the field. To reduce the chance of missing relevant studies, we also consulted a health sciences librarian about our search strategy. The full search strategy for each database can be found in Appendix S2 in supporting information.

### 2.2 Screening

The publications were imported to Rayyan for screening,^14^ and duplicate publications were removed. One author (K.T.) then screened the retrieved publications by title and abstract using predetermined inclusion and exclusion criteria. The results were cross‐examined by another author (S.G.), and disagreements were resolved through discussion. Included studies met the following criteria: (1) full text written in English, (2) focus on unpaid caregivers of people with dementia and an age‐related hearing and/or vision impairment. Publications that focused exclusively on paid caregivers (e.g., health‐care providers) were excluded. Given the National Library of Medicine's definition of dementia as “an acquired organic mental disorder with loss of intellectual abilities of sufficient severity to interfere with social or occupational functioning,”^15^ we included dementia due to various reasons, whether self‐identified by the patient or a proxy or diagnosed by a professional. We included all types of hearing and vision impairments if they were age‐related (i.e., samples with individuals born deaf were excluded). Publications that did not focus on individuals with both a cognitive impairment and either an age‐related hearing impairment or an age‐related vision impairment were excluded. Publications were also excluded if they were not empirical studies (e.g., reviews, opinion pieces, study protocols, book chapters). The remaining publications were then screened by full text according to the same inclusion and exclusion criteria, resulting in a final sample of 12 articles for review.

### 2.3 Data extraction and synthesis

The 12 articles were then coded by publication year, country, study aims, research method, sample characteristics, key findings, and implications. Quantitative findings were examined and interpreted using narrative synthesis to gain an understanding of the experiences of unpaid caregivers of people with dementia and concurrent age‐related hearing or vision impairment, as well as methodological trends and limitations in the literature. Qualitative findings were synthesized using applied thematic analysis to arrive at the main themes reported in this article.^16^

We also assessed the levels of evidence reported in the final 12 publications. To assess studies with a mixed methods design, we used McGill University's Mixed Methods Appraisal Tool.^17^ For all other study designs, we used the critical appraisal tools developed by the Joanna Briggs Institute.^18^, ^19^, ^20^, ^21^ To compute scores for the quality of each study, questions answered with “yes” received 1 point, and questions answered with “no” or “unclear” received no point. The total score was then divided by the number of questions and multiplied by 100. Articles were rated as very poor (0–30%), poor (31%–50%), fair (51%–70%), good (71%–90%), or excellent (> 90%). Two reviewers (S.G., K.T.) independently completed the critical appraisal for each study; their interrater reliability before reconciliation reached 98.1%; all disagreements were resolved through discussion.

## 3 RESULTS

### 3.1 Search and screening

**FIGURE 1:**
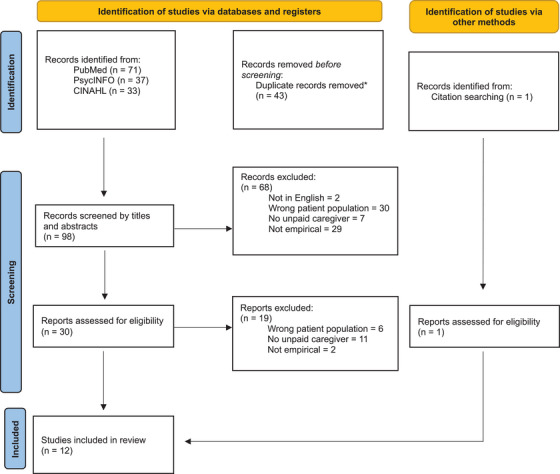
Preferred Reporting Items for Systematic Reviews and Meta‐Analyses (PRISMA) 2020 flow diagram for new systematic reviews which included searches of databases, registers, and other sources. *Removed by automation tool. From ref. 12.

The initial keyword search of the three databases yielded 141 publications (71 from PubMed, 37 from PsycINFO, and 33 from CINAHL). When these publications were imported to Rayyan, 43 duplicates were identified and removed; a total of 98 non‐duplicate publications remained for screening of titles and abstracts. A total of 68 publications were thus excluded, resulting in 30 publications for full‐text review. Finally, after screening by full text, 19 publications were excluded; a total of 11 remained. One additional publication was added via citation searches of the included studies, for a final sample of 12 publications. Figure 1 presents details of the full search and screening process.

A summary of the 12 studies is presented in Table 1.^5^, ^6^, ^22^, ^23^, ^24^, ^25^, ^26^, ^27^, ^28^, ^29^, ^30^, ^31^ Key descriptive characteristics of the 12 studies can be found in Table 2.^5^, ^6^, ^22^, ^23^, ^24^, ^25^, ^26^, ^27^, ^28^, ^29^, ^30^, ^31^ The studies were published from 2009 to 2023, with the majority (58%) published in the last 5 years. They were conducted in five different countries: the UK (*n* = 6; 50%), the United States (*n* = 4; 33%), France (*n* = 4; 33%), Cyprus (*n* = 3; 25%), and Australia (*n* = 1; 8%). Three studies^5^, ^26^, ^31^ were conducted in the UK, France, and Cyprus, so they are accounted for multiple times; these studies were part of SENSE‐Cog, a European‐wide project focused on exploring the impact of dementia and concurrent age‐related hearing and vision impairment.^32^

**TABLE 1 alz70525-tbl-0001:** Key descriptive characteristics of the 12 studies in the final sample.

Characteristic (*N* = 12)	Frequency, *n* (%)
**Year of publication**	
2012 and earlier	1 (8)
2013–2018	4 (33)
2019–2024	7 (58)
**Country of publication** ^a^	
United Kingdom	6 (50)
United States	4 (33)
France	4 (33)
Cyprus	3 (25)
Australia	1 (8)
**Method**	
Qualitative	4 (33)
Randomized control trials	2 (17)
Quasi‐experimental	2 (17)
Cross‐sectional	2 (17)
Mixed methods	2 (17)
**Vision versus hearing impairment**	
Hearing	5 (42)
Vision	4 (33)
Both	3 (25)

^a^ Studies were accounted for multiple times if they met criteria.

**TABLE 2 alz70525-tbl-0002:** Summary of the 12 studies in the final sample.

Author	Year	Country	Study aim	Method	Caregivers	Patients	Key findings	Implications
Lawrence et al.^25^	2009	UK	To explore the experiences and needs of older adults with VI and dementia.	Qualitative (interviews)	17 family caregivers (10 children)	17 older adults with VI (via Snellen acuity, Seeing Severity Scale) and dementia (via MMBlind, IQCODE, and the Clinical Dementia Rating scale) living in community and residential settings	Concerns related to PwD's safety led the family caregiver to restrict their relative's activity, which led to conflict; dependent on family caregivers for orientation, which led to caregiver physical exhaustion; family caregivers unprepared to deal with PwD's hallucinations; family caregivers felt burden of providing social support to PwD who often experience loneliness and isolation; activities (e.g., day centers and lunch clubs) were praised by family caregivers.	Need increased education to family caregivers on dealing with hallucinations; need further research to identify the prevalence of VI in dementia care homes.
Adrait et al.^22^	2017	France	To assess the efficacy of hearing aids for patients with HL and AD.	RCT	Informal caregivers living with the older adult.	48 community‐dwelling individuals with probable diagnosis of AD (via DSM IV, NINCDS‐ADRDA, MMSE) and bilateral sensorineural HL (via pure tone audiometry)	No evidence that hearing aids improve QoL of hearing‐impaired AD patients or their informal caregivers after 6 months of intervention.	Need to include younger participants at earlier stages of the disease.
Bunn et al.^23^	2017	UK	To explore the impact of comorbidities (including VI) on access to non‐dementia services and continuity of care.	Qualitative (interviews, focus groups; thematic content analysis was informed by theories of continuity of care and access to care)	33 family caregivers (64% spouse)	28 community‐dwelling PwD (AD, mixed, vascular, or with Parkinson's disease) and a comorbidity (24% VI; 17% diabetes and VI; 34% diabetes, stroke, and VI) as reported by HCP	Family members serve essential role for PwD by advocating and navigating the health‐care system, but no formal integration of family caregiver into care planning or formal support; availability of a family caregiver impacts PwD's access to care; challenges of family caregivers: understanding health‐care system, their own physical and emotional health, personal responsibilities; PwD and family caregivers’ value continuity in care.	Need interventions to promote partnership working (between clinicians, family caregivers, and PwD) and tailored care for PwD and VI. Need to enhance communication and collaboration across specialties, services, and family caregivers to provide integrated care for PwD and VI.
Mamo et al.^6^	2017	USA	To test the feasibility of a basic, low‐cost hearing intervention for PwD.	Quasi‐experimental (community‐based participatory research; social cognitive theory; human factors approach to design)	20 caregivers (40% spouse, 25% adult children)	20 PwD (via MMSE) and HL (via test frequencies or tones)	Caregivers reported satisfaction with the intervention; no significant changes in caregiver burden (possibly increased short‐term due to challenges learning a new technology; measuring frequency of use of the low‐cost hearing intervention relied on the caregiver vs. traditional hearing aids, which automatically track hours of use).	Need to target patients with higher symptom burden at baseline (greater benefit from the intervention); need to incorporate specific questions about source of burden.
Nyman et al.^28^	2017	UK	To explore the social care and support needs of PwD and VI, as well as the barriers/ facilitators for meeting these needs.	Qualitative (interviews)	12 spouses, 3 relatives	25 PwD and concurrent VI residing in community‐based housing (self‐reported)	Concurrent presence of dementia and VI can exacerbate existing difficulties and increase dependence; important to stay active (physically and socially), but requires caregiver assistance; frequently use assistive technology (e.g., mobility devices and visual devices), but barriers to learning technology, some are not usable due to dual impairment (e.g., can't see memory aids or can't concentrate on VI aids), and they are expensive to acquire; familiarity was a facilitator of independence (e.g., remain in their home or bring own furnishings to assistive living, continuous paid caregiver).	Need to promote early visual health assessments of PwD to facilitate timely education and medical services.
Jorgensen et al.^24^	2019	USA	To explore if caregivers accept TeleCare, test whether it reduces their stress and burden, and determine whether it improves communication with patients.	RCT	20 caregivers skilled in using a smart phone	20 PwD (via MMSE ≤ 28 and own power of attorney) and bilateral HL to warrant amplification (via audiogram)	Caregivers reported low burden; TeleCare significantly reduced the stress caregivers reported feeling due to balancing caring for the patient and meeting their other personal responsibilities; caregivers accepted the intervention; communication goals improved.	Need to include participants with a range of cognitive decline (the included patients all had mild cognitive decline, which may have reduced study effects).
Wolski et al.^31^ (SENSE‐Cog)	2019	UK, France, Cyprus	To explore the challenges in conducting visual, hearing, and cognitive assessments for individuals with dual or triple impairments.	Qualitative (focus groups, interviews)	10 spouses, 4 close family members	18 PwD (via established diagnosis; mild‐to‐moderate; due to AD, vascular dementia, or mixed) and self‐reported HL or VI	The assessments did not increase the care partners’ understanding or ability to cope with the PwD; care partners rely on their own research regarding the cognitive and sensory conditions; caregivers stressed the importance of knowing their clinicians and continuity of care; in privatized systems (e.g., Cyprus), there are added challenges of accessing multidisciplinary care and health information; in socialized systems (e.g., UK), accessing care was easier, but tended to be fragmented.	Need to improve care partner education on technical devices and disorders; need for better inclusion of care partner; need for better collaboration between specialists.
Leroi et al.^26^ (SENSE‐Cog)	2020	France, UK, Cyprus	To evaluate the effectiveness of a sensory intervention on dementia‐related outcomes.	Quasi‐experimental (intervention based on the Behavior Change Wheel)	19 unpaid caregivers (family or friends)	19 community‐based PwD (via formal diagnosis of early–moderate (MoCA ≥ 24) AD, vascular dementia, or mixed dementia) and adult‐acquired mild‐to‐moderate bilateral HL (via HearCheck screening device) and/or VI (via PEEK tool)	Feasibility of intervention was previously supported; basic intervention (i.e., assessments tailored to PwD and fitting of lenses/hearing aids) led to slight decrease in relationship satisfaction (high at baseline), small deterioration in physical well‐being from baseline, average time spent assisting with personal ADLs increased by 17 hours/month, but time spent assisting with instrumental ADLs decreased by 22 hours/month and time spent supervising declined by 39 hours/month, qualitative findings demonstrated positive effects; extended intervention (i.e., basic plus support from a sensory support therapist) led to notable improvements in PwD's hearing, which reduced dependence on caregivers, improved communication, and improved relationship quality for the dyad.	Need RCT to confirm the effectiveness of the sensory support intervention. Need to explore the broader impacts of sensory support interventions on dementia‐related outcomes, including behavioral disturbances and social engagement.
Meyer et al.^27^	2020	Australia	To evaluate the feasibility of “Hear‐Communicate‐Remember,” a telehealth‐delivered intervention for caregivers conducted by a speech–language pathologist, audiologist, or psychologist.	Mixed method (survey, interviews; intervention based on the Behavior Change Technique Taxonomy)	5 spouses, 1 daughter	6 community‐dwelling PwD and HL (self‐reported)	Caregivers found the intervention helpful and appreciated they could return to the videos to refresh their memory; originally intended to be telehealth‐delivered, but technical difficulties required more in‐person sessions; differing opinions on appropriateness of telehealth—some thought it increased access, but others missed the human contact and were worried about poor technology literacy and internet access; caregivers reported increased knowledge to improve hearing aid use and communication and memory strategies; caregivers reported implementing these strategies into their daily lives; caregivers reported increased communication, improved psychosocial health of PwD, and improvements to their own mental health (e.g., feeling less stressed); increased hearing aid use.	Intervention should be delivered at earlier stages of dementia to maximize positive impact; need to use more suitable technology; need to measure the type and degree of dementia and HL.
Varadaraj et al.^30^	2020	USA	To gain understanding about caregiving for PwD and VI.	Cross‐sectional (secondary data analysis – 2011 NHATS & NSOC)	1776 unpaid caregivers (53% child)	1196 community dwelling Medicare beneficiaries (65+) with self‐reported VI and probable or possible dementia based on self‐reported physician diagnosis of dementia or AD, Informant Interview to Differentiate Aging and Dementia score, or cognitive performance tests	Caregivers of PwD and VI were less likely to be a spouse, more likely to be an adult child, more likely to be providing care for > 4 years, more likely to assist with mobility, banking, self‐care activities, and health management, reported 3.2 times as many personal valued activities were affected per month, and spent 1.7 times more hours on caregiving than caregivers of people without either impairment; caregivers of PwD or VI spent 1.3 times more hours and had 1.3–1.9 times as many valued activities affected compared to caregivers of those without impairments, suggesting additive implications.	Need for longitudinal research to determine causal relationships and impact of time.
Leroi et al.^5^	2022	France, UK, Cyprus	To explore the unmet support care needs of people with HL and/or VI in PwD and their care partners.	Mixed methods (survey, focus groups, interviews)	97 care partners (62.5% spouses; purposive sub‐sample of 34 dyads participated in focus groups or interviews)	97 community‐dwelling PwD (60+ with diagnosis of dementia in mild‐moderate stage; IQCODE) and HL and/or VI severe enough to impact daily life (HHIE‐S, LVVFQ)	Care partners reported greater stress/burden as severity of impairments increased; multi‐morbidity exacerbates potential for conflict with PwD; care partners reported feeling inadequate, helpless, and overwhelmed; care partners reported being overprotective, which increases dependency of PwD and further decreases their functional abilities, and in turn, adds to caregiver burden; negative impact on care partner's mental and physical health; care partners reported feeling misunderstood by HCPs and lack of knowledge/skills for using corrective devices.	Need for supportive interventions aimed at alleviating loneliness and social isolation (on both the PwD and their care partners); need for improved communication with and between HCPs (especially because needs span multiple disciplines).
Powell et al.^29^	2023	USA	To examine how HL and dementia influences communication with HCPs and the role of caregivers during medical visits.	Cross‐sectional (secondary data analysis – 2015 NHATS)	2631 unpaid care partners	7070 community‐dwelling older adults (65+) with and without dementia or HL (self‐reported)	70% of PwD were accompanied to medical visits by a caregiver, compared to 75% of PwD and HL, 23% with HL alone, and 17% with neither condition; presence of HL and dementia was associated with caregivers assuming a more active role in medical visits.	Need for evidence‐based inclusion of caregivers in the care team (cost saving potential); need technology‐based interventions to educate caregivers on patient advocacy, communication skills, decision making, and care planning; need to use objective hearing measures and consider dementia severity.

Abbreviations: ADL, activities of daily living; AD, Alzheimer's disease; ADRD, Alzheimer's disease or related dementias; DSM IV, Diagnostic and Statistical Manual of Mental Disorders, 4th Edition; HCP, health‐care provider; HHIE‐S, Hearing Handicap Inventory for the Elderly Screening Version; HL, hearing loss; IQCODE, Informant Questionnaire on Cognitive Decline in the Elderly; LVVFQ, Low Vision Visual Functioning Questionnaire; MMBlind, Mini‐Mental State Examination for the visually impaired; MMSE, Mini‐Mental State Examination; MoCA, Montreal Cognitive Assessment; NINCDS‐ADRDA, National Institute of Neurological and Communicative Disorders and Stroke–Alzheimer's Disease and Related Disorders Association; NHATS, National Health and Aging Trends Study; NSOC, National Study of Caregiving; PCA, posterior cortical atrophy; PwD, people with dementia; QoL, quality of life; RCT, randomized controlled trial; VI, vision impairment.

### 3.2 Study designs, instruments, and theoretical frameworks

The most common research design was qualitative (*n* = 4; 33%), and these studies were conducted with interviews and focus groups for data collection. The remaining studies were randomized control trials (*n* = 2; 17%), quasi‐experimental (*n* = 2; 17%), cross‐sectional (*n* = 2; 17%), or mixed methods (*n* = 2; 17%). All of these studies relied on questionnaires for data collection. The two studies with a mixed methods design included interviews and/or focus groups, along with questionnaires.^5^, ^27^

Meyer et al.^27^ developed a 7‐item satisfaction survey for their study but did not report psychometrics. Two studies^6^, ^22^ used the Zarit Burden Interview, which is widely used for assessing burden in caregivers of people with dementia; this instrument has good internal consistency reliability (Cronbach alpha 0.92).^33^ Two studies^5^, ^26^ assessed caregivers’ mental health using the validated and reliable Geriatric Depression Scale‐15 and the Patient Health Questionnaire‐15. Jorgensen et al.^24^ adapted the Burden Scale for Family Caregivers to form their own 11‐question 5‐point burden scale; although the original survey has established reliability (Cronbach alpha 0.90–0.91) and validity,^34^ the authors did not report psychometrics for their adapted instrument. Leroi et al.^5^ used the short version of the Burden Scale for Family Caregivers (Cronbach alpha 0.92).^35^ Leroi et al.^5^ adapted the 59‐item Supportive Care Needs Survey (Cronbach alpha 0.87–0.97)^36^ to assess the needs of people with dementia with hearing and/or vision impairment; the authors discussed efforts to retain content validity, but they did not report psychometrics for their adapted questionnaire.

Other instruments in the studies, all with established reliability, were the Family Care Giving Role Scale,^37^ as well as the Relationship Satisfaction Scale;^26^ the International Outcome Inventory‐Alternative Intervention‐Significant Other;^6^ and the Duke Health Profile centered on patient and caregiver social interactions.^22^ Finally, the two cross‐sectional studies used secondary data analysis to assess findings from the National Health and Aging Trends Study^29^, ^30^ and the National Study of Caregiving.^30^ Many of the studies also assessed patient outcomes; we do not report these outcomes, because our focus is on caregivers.

Four of the included studies were guided by a theoretical framework; the remaining (*n* = 8; 67%) lacked theoretical guidance. Mamo et al.^6^ used community‐based participatory research, drawing on social cognitive theory and a human factors approach to design. Leroi et al.^26^ used the Behavior Change Wheel, and Meyer et al.^27^ used the Behavior Change Technique Taxonomy. The qualitative study by Bunn et al.^23^ was informed by theories of continuity of care and access to care.[Table alz70525-tbl-0001], [Table alz70525-tbl-0002]

### 3.3 Participants

All 12 studies focused on caregivers of people with dementia and sensory impairments. Five (42%) focused on hearing impairments, four (33%) on vision impairments, and the remaining included people with hearing and vision impairments (*n* = 3; 25%). Besides one study that included patients living in residential care settings,^25^ the remaining 11 studies focused exclusively on community‐dwelling people with dementia. The sample sizes of patients ranged from 6 to 7070; half of the studies (*n* = 6; 50%) included ≤ 20 patients. The sample sizes of caregivers ranged from 6 to 2631; one study by Adrait et al.^22^ did not report sample size, and a majority of those that did (*n* = 7; 58%) included ≤ 20 caregivers. Four studies^22^, ^24^, ^26^, ^29^ did not specify how the caregivers were related to the patients, six included primarily the spouses of the patients,^5^, ^6^, ^23^, ^27^, ^28^, ^31^ and two included primarily the children of the patients.^25^, ^30^ Three studies included both unpaid and paid caregivers in their samples.^23^, ^25^, ^28^

Across seven of the studies, the following instruments were used to assess dementia: the Mini‐Mental State Examination;^6^, ^22^, ^24^ the Clinical Dementia Rating scale, the Montreal Cognitive Assessment‐BLIND, and the Informant Questionnaire on Cognitive Decline in the Elderly;^5^, ^25^ the Montreal Cognitive Assessment;^26^ and the Diagnostic and Statistical Manual of Mental Disorders and the National Institute of Neurological and Communicative Disorders and Stroke and the Alzheimer's Disease and Related Disorders Association criteria.^22^ Three studies^6^, ^22^, ^24^ used a hearing test for hearing impairment, and one study^25^ used the Snellen chart and the Seeing Severity Scale for vision impairment. One study^26^ used HearCheck, a handheld screening device, and PEEK, a smartphone visual acuity app, to screen for impairments. One study^23^ relied on a health‐care–reported diagnosis of dementia and vision impairment. Leroi et al.^5^ used the Hearing Handicap Inventory for the Elderly Screening Version and the Low Vision Visual Functioning Questionnaire. The remaining five studies^27^, ^28^, ^29^, ^30^, ^31^ relied on self‐reported diagnoses of dementia, hearing, and/or vision impairment.

### 3.4 Caregiver burden

The studies in this review found that caring for people with dementia and sensory impairments places substantial demands on caregivers, often leading to strain on their personal time and responsibilities. Multiple studies in this review highlight the significant time commitment required in such caregiving roles.^26^, ^30^ For instance, Varadaraj et al.^30^ found that caregivers of people with both dementia and vision impairment spent 1.7 times more hours providing care compared to caregivers of individuals without cognitive or visual impairments. Furthermore, their personal activities were 3.2 times more likely to be disrupted each month. Even when only one impairment—either cognitive or visual—was present, caregivers still experienced elevated caregiving demands, spending 1.3 times more hours and facing 1.3 to 1.9 times more disruptions to their personal lives, compared to caregivers of people without sensory impairments.^30^ These findings suggest that co‐existing impairments result in an additive caregiving burden.

Qualitative data reinforce these quantitative findings. In a study by Leroi et al.,^5^ caregivers described increasing levels of stress and burden as the severity of impairments worsened. Multi‐morbidity was described as intensifying daily caregiving challenges and exacerbating the potential for conflict with care recipients. In addition to the time required for hands‐on care, caregivers often provide essential support for orientation and social interaction, responsibilities that extend beyond basic caregiving tasks and impact their physical and emotional health.^5^, ^23^, ^25^, ^28^, ^30^ Notably, Lawrence et al.^25^ highlighted that family caregivers frequently felt the burden of having to alleviate the loneliness and social isolation experienced by people with dementia. Caregivers emphasized the positive impact of structured community‐based programs, such as day centers and lunch clubs, which provide meaningful engagement for care recipients and temporary relief for caregivers.

Similar challenges are reported when caring for people with dementia and hearing impairment. For example, Powell et al.^29^ found that 75% of people with both dementia and hearing impairment were accompanied by a caregiver to medical appointments. This rate was higher than that of people with dementia alone (70%), hearing impairment alone (23%), or neither impairment (17%). This suggests that dual impairments necessitate more intensive caregiver involvement, including in health‐care settings. Moreover, caregivers of those with both impairments often assume more active roles in medical visits.

On the other hand, Jorgensen et al.^24^ found that caregivers of individuals with mild cognitive decline and hearing impairment reported experiencing stress related to balancing caregiving responsibilities with work and family obligations. Notably, despite these challenges, the caregivers indicated a relatively low overall caregiving burden, as measured by a general burden scale commonly used for severe illnesses or life circumstances. This lower burden may be attributed to the participants’ relatively mild cognitive impairment and their status as first‐time hearing aid users. These findings suggest that even when the overall perceived burden is low—particularly in cases involving mild impairments—the competing demands of caregiving can still lead to considerable tension and stress.

### 3.5 Unmet caregiver needs and potential strategies

Overall, the studies illuminated several unmet needs of caregivers and potential strategies for addressing those needs. One prominent theme was the challenges caregivers face in navigating complex health‐care systems. Bunn et al.^23^ noted that despite family caregivers being essential to the health and well‐being of people with dementia and visual impairment, there is no formal integration of family caregivers into care planning. Three other studies of people with cognitive and sensory impairments supported this finding, reporting a need for better inclusion of the care partner within the care team.^5^, ^29^, ^31^ This exclusion not only limits caregivers’ ability to advocate effectively for their loved ones but may also hinder efficient care delivery. Some evidence suggests that better inclusion of care partners might even contribute to health‐care cost savings.^29^

Beyond the need for formal inclusion, caregivers consistently emphasized the importance of continuity of care and strong relationships with health‐care providers.^23^, ^28^, ^31^ For instance, in a cross‐national study, caregivers in privatized health‐care systems (e.g., Cyprus) faced challenges in accessing multidisciplinary care and health information, whereas in socialized health‐care systems (e.g., the UK), care was more accessible but often fragmented, leading to caregiver frustration over poor collaboration between specialists.^31^ Similarly, participants in Leroi et al.^5^ stressed the need for better communication between providers, particularly when multiple specialists were involved in managing co‐existing cognitive and sensory impairments.

Another frequently cited gap was the lack of education and training for caregivers. In five studies, caregivers expressed a need for better guidance on how to manage people with both dementia and sensory impairments.^5^, ^25^, ^28^, ^29^, ^31^ For instance, one study^31^ mentioned that because there were no sufficient instructions, many caregivers resorted to independent research to meet their loved ones’ care needs. This knowledge gap was particularly evident in the context of assistive technologies. Although such tools are commonly used to support individuals with visual impairment, caregivers reported that they were often difficult to operate and prohibitively expensive.^28^ Two other studies similarly noted a need for increased caregiver education on how to use technical devices for individuals with cognitive, hearing, and visual impairments.^5^, ^31^ The effectiveness of such technology was also questioned; for example, Nyman et al.^28^ noted that memory aids may be visually inaccessible to those with visual impairment, while visual aids may be ineffective for individuals who struggle with cognitive decline.

### 3.6 Interventions to address caregiver burden

A number of interventions aimed at supporting caregivers of individuals with dementia and sensory impairments have been tested.^6^, ^22^, ^26^, ^27^ These interventions varied in scope, delivery format, and measured outcomes, and their overall impact on caregiver burden was mixed.

The interventions tested included hearing aids;^22^ education and an over‐the‐counter, low‐cost amplification device;^6^ TeleCare, a primarily telehealth‐delivered intervention including a hearing aid fitting, monitoring, education, and support;^24^ hearing aid and communication strategy education delivered via video and face‐to‐face sessions;^27^ and a vision and hearing assessment, tailored to people with cognitive impairment, fitting of lenses or hearing aids, support, education, and supplementary sensory aids.^26^

Hearing aids showed modest potential for reducing caregiver burden. In a randomized controlled trial, Adrait et al.^22^ tested the effectiveness of traditional hearing aids in individuals with Alzheimer's disease and hearing impairment. After 6 months, there was no significant improvement in caregiver quality of life. In another study, Mamo et al.^6^ evaluated a low‐cost, over‐the‐counter hearing amplification device, alongside caregiver education. Although caregivers reported satisfaction with the intervention, it did not result in a significant reduction in caregiver burden. The authors even hypothesized that caregiver burden may have temporarily increased due to the learning curve associated with the new technology. In addition, unlike conventional hearing aids, which automatically log usage, the device required caregivers to manually track use, adding to their responsibilities.

A broader sensory intervention was studied by Leroi et al.,^26^ who implemented a program including vision and hearing assessments tailored to people with dementia and fittings for lenses and hearing aids. While the intervention was found to be feasible and qualitatively positive, it did not result in significant improvements in caregiver reported relationship satisfaction or physical well‐being. Interestingly, the intervention had mixed effects on caregiving time. On average, caregivers spent 17 additional hours per month assisting with personal activities of daily living, but this increase was offset by a reduction of 22 hours assisting with instrumental activities of daily living and 39 hours in supervision per month. These shifts suggest a redistribution of caregiving responsibilities, possibly reflecting care recipients’ increased independence and reduced caregiver burden in specific domains. Notably, when the intervention was extended to include support from a sensory support therapist, caregivers observed a meaningful decrease in care recipient dependence, along with improvements in communication and relationship quality. This was the only study in our review that evaluated the effectiveness of a sensory intervention for visual impairment.

Two studies^24^, ^27^ in our review consisted of a virtual intervention for caregivers of people with dementia and hearing impairment. Jorgensen et al.^24^ tested “TeleCare,” which consisted of a bilateral hearing aid fitting, remote monitoring of wearing behaviors and patient satisfaction, at‐home programming, and real‐time video calls with providers. This intervention led to significant reductions in caregiver stress, increased communication between caregivers and patients, and was well accepted by the caregivers. Similarly, Meyer et al.’s^27^ developed the “Hear–Communicate–Remember” program, which delivered four weekly modules to caregivers via telehealth. These sessions, facilitated by a speech–language pathologist, audiologist, or psychologist, focused on hearing aid support, memory strategies for hearing aid use, and communication strategies. Although this intervention was intended to be delivered entirely via telehealth, technical difficulties required several sessions to be delivered in person. Caregivers disagreed about the appropriateness of telehealth: some thought that it increased access to the intervention, and they appreciated that they could return to the videos to refresh their memory; others were worried about technical difficulties or having reliable access to the internet. The caregivers gained knowledge from the intervention and reported that they implemented the learned communication and memory strategies in their daily lives, which they said helped to increase hearing aid use and communication. In addition, the caregivers reported feeling less stressed, with positive changes in their psychosocial well‐being.

### 3.7 Methodological considerations

Several methodological considerations and areas for future inquiry were noted across the 12 studies. First, several studies called for improved reporting on types and degrees of impairments, as well as samples with a wider variety of impairment acuities. In two of the studies,^27^, ^29^ the authors recommended using objective hearing measures and considering the degree of dementia severity, because these might influence study results. Adrait et al.^22^ and Meyer et al.^27^ recommended including younger participants at earlier stages of disease to maximize the positive impact of hearing aids; however, Mamo et al.^6^ found the opposite: patients with higher symptom burden at baseline had the greatest benefits from hearing amplification. Jorgensen et al.^24^ stated that their modest study effects on caregiver burden might have been due to participants’ mild cognitive impairment. They suggested that future studies include participants with a range of cognitive decline. Another study identified the need for future research to establish the prevalence of vision impairments in dementia care homes.^25^ Veradaraj et al.^30^ emphasized the need for longitudinal research to determine causal relationships and the influence of time.

### 3.8 Quality assessment

The results of the quality assessment are presented in Appendix S3 in supporting information. Four publications (33%) were rated excellent,^5^, ^23^, ^29^, ^30^ four were rated good (33%),^22^, ^27^, ^28^, ^31^ three were rated fair (25%),^6^, ^25^, ^26^ and one was rated poor (8%).^24^ The four qualitative studies^23^, ^25^, ^28^, ^31^ lacked philosophical and theoretical guidance. They also lacked adequate discussion of the researchers’ impact on their research. The two quasi‐experimental studies^6^, ^26^ were rated fair due to a lack of control groups, multiple measurements, and adequate follow‐up. The randomized controlled trial by Jorgensen et al.^24^ was rated poor due to a general lack of empirical reporting; however, we kept this study in our sample because it was one of only two randomized controlled trials, and it provided important findings. Although the majority of publications were rated good or better, there is a need for more rigorous study.

## 4 DISCUSSION

The findings from this review highlight the complex, multifaceted challenges faced by caregivers of people with dementia who also have hearing and/or vision impairments. Among the 12 reviewed studies, qualitative methods were predominant, relying on interviews and focus groups to gather detailed insights from caregivers. The remaining studies used randomized control trials, quasi‐experimental designs, cross‐sectional designs, and mixed method designs, relying primarily on questionnaires for data collection. Caregivers of individuals with both cognitive and sensory impairments reported significantly higher levels of burden than did those caring for individuals with either dementia or sensory impairment alone.

Caregiver burden emerged as a critical issue, consistent with previous literature.^1^, ^7^, ^8^, ^9^ Studies have shown that caregivers of people with dementia and sensory impairment spend significantly more time on caregiving duties, which affects their personal responsibilities and activities. This review corroborates those findings, illustrating that the additive effect of single or dual impairments exacerbates the caregiving burden. Unique to caregivers of dementia patients with sensory impairments is the increased need for orientation and social support, further taxing their physical and emotional health.^23^, ^25^, ^28^ The increased caregiving hours and affected personal activities highlight the compounded challenges faced by these caregivers.

Unmet needs among caregivers of dementia patients with sensory impairments are a significant concern. Our review identified several critical areas, including difficulties in navigating the health‐care system, insufficient inclusion of care partners in the care team, challenges in accessing multidisciplinary care, a lack of education on managing dementia with sensory impairments, and the high cost of assistive technologies. Previous studies have also highlighted these gaps, emphasizing the need for comprehensive support systems for dementia caregivers in general.^38^ Additional needs specific to these caregivers include tailored training programs to manage sensory impairments and improved access to affordable assistive devices. Future studies should continue to explore the feasibility and effectiveness of using technology‐assisted devices to develop tailored person‐centered care strategies to meet the unique needs of caregivers of dementia patients with sensory impairment.

Meanwhile, interventions to assist caregivers of people with dementia and sensory impairments have shown varying degrees of effectiveness. Hearing aids, for instance, have demonstrated modest potential in reducing caregiver burden when combined with visual and hearing assessments tailored to the needs of dementia patients.^26^ Virtual interventions also present promising alternatives, evidenced in studies of online support for caregivers; they have been effective in providing accessible, flexible resources.^24^, ^27^ These interventions highlight the importance of integrating technology and personalized care strategies to support caregivers.

Future research should focus on several key areas to further understand and support this caregiver population. First, researchers need to explore the long‐term effectiveness of various interventions, particularly those involving accessible technology and personalized care strategies. Second, there is a need to address systematic causes of caregiver burden. Researchers should work to improve collaboration between specialists and caregivers, as well as increase caregiver support and education. Interventions coupled with health‐care provider support will maximize effectiveness and improve the quality of life for both the patient and the caregiver. Third, there is a need to investigate the specific experiences and challenges faced by caregivers of individuals with dual sensory impairments, to develop targeted support mechanisms. Researchers should also consider how caregivers’ needs may change depending on whether the older adult is living in the community or residing in a long‐term care facility. Caregivers in Nyman et al.^28^ emphasized the importance of maintaining environmental familiarity for individuals with dementia and visual impairments, noting that aging in place—or replicating the home environment in care settings by bringing familiar furnishings—can promote independence and reduce disorientation. Future studies might explore how environmental stability can be leveraged as a low‐cost, caregiver‐identified strategy to support both patients and their care partners. Finally, theory‐driven research is necessary; only four of the reviewed papers were explicitly guided by a theoretical framework. Addressing the diverse, evolving needs of caregivers requires ongoing research to fill gaps and inform policy and practice.

Of the seven papers that provided the caregivers’ relationship to the patient, five mostly involved the patients’ spouses. Because hearing and vision impairments become more common with age, these caregivers are at risk of sensory impairments themselves. As the population of older adults continues to grow, coupled with worldwide care worker shortages, the challenges of spousal caregivers will be further exacerbated.^39^, ^40^, ^41^ In a study^42^ with visually impaired informal caregivers, challenges similar to those experienced by non‐visually impaired caregivers have been discussed; however, these caregivers faced additional burdens such as transportation difficulties. The authors emphasized the need to develop interventions adapted for visually impaired caregivers. Similarly, two studies^43^, ^44^ of deaf informal caregivers of people with dementia have noted the unique challenges faced by this population, including safety concerns. Ammons et al.^43^ emphasized the need for accommodations in mainstream support groups to promote these caregivers’ participation, as well as accessible medical and nursing care education on how to maintain quality of life and provide the best care possible to their loved ones. These caregivers did not have age‐related hearing impairment, and the populations of the deaf and those with age‐related hearing impairment face different challenges. Although this was not the focus of the present literature review, future research must address the unique needs of caregivers who themselves have or are at risk of developing a sensory impairment.

### 4.1 Limitations

This systematic review has certain limitations. First, we searched only three electronic databases and included only publications written in English. Second, our search terms were not exhaustive; but we did consult a health sciences librarian about our search strategy, and we searched the citations of our final sample to reduce chances of missing important studies. Finally, the diversity in study designs and methodologies among the included studies may have introduced variability in the findings.

## 5 CONCLUSION

Caregivers of individuals with dementia and sensory impairments face substantial burdens and unmet needs. Policy makers and health‐care providers should consider implementing comprehensive support systems that address those unique needs. This will include enhancing caregiver education, improving access to multidisciplinary care, and making assistive technologies more affordable and accessible. By acknowledging and addressing these challenges, we can improve the quality of life for both caregivers and care recipients. Tailored interventions and comprehensive support systems will then alleviate caregiver strain and improve outcomes. In these ways, we can better support caregivers and enhance the care provided to individuals with dementia and sensory impairments.

## CONFLICT OF INTEREST STATEMENT

The authors declare no conflict of interests. The author declarations are available in supporting information.

## Supporting information



Supporting Information

Supporting Information
