# Supplementary material for: Informal caregiving for people with dementia and hearing or vision impairment: A systematic review
Source: Alzheimers Dement. 2025 Aug 14;21(8):e70525. doi: 10.1002/alz.70525 (PMC12351394; doi:10.1002/alz.70525)
Supplement: Supplementary file 1 — Supporting Information [file ALZ-21-e70525-s002.docx]

Appendix 1. PRISMA Checklist


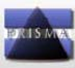
 PRISMA 2020 Checklist

| **Section and Topic** | **Item #** | **Checklist item** | **Location where item is reported** |
| --- | --- | --- | --- |
| **TITLE** | | |  |
| Title | 1 | Identify the report as a systematic review. | Title |
| **ABSTRACT** | | |  |
| Abstract | 2 | See the PRISMA 2020 for Abstracts checklist. | Abstract |
| **INTRODUCTION** | | |  |
| Rationale | 3 | Describe the rationale for the review in the context of existing knowledge. | Introduction |
| Objectives | 4 | Provide an explicit statement of the objective(s) or question(s) the review addresses. | Introduction |
| **METHODS** | | |  |
| Eligibility criteria | 5 | Specify the inclusion and exclusion criteria for the review and how studies were grouped for the syntheses. | 2.2 Screening |
| Information sources | 6 | Specify all databases, registers, websites, organisations, reference lists and other sources searched or consulted to identify studies. Specify the date when each source was last searched or consulted. | 2.1 Search |
| Search strategy | 7 | Present the full search strategies for all databases, registers and websites, including any filters and limits used. | Appendix B |
| Selection process | 8 | Specify the methods used to decide whether a study met the inclusion criteria of the review, including how many reviewers screened each record and each report retrieved, whether they worked independently, and if applicable, details of automation tools used in the process. | 2.2 Screening |
| Data collection process | 9 | Specify the methods used to collect data from reports, including how many reviewers collected data from each report, whether they worked independently, any processes for obtaining or confirming data from study investigators, and if applicable, details of automation tools used in the process. | 2.3 Data extraction/synthesis |
| Data items | 10a | List and define all outcomes for which data were sought. Specify whether all results that were compatible with each outcome domain in each study were sought (e.g. for all measures, time points, analyses), and if not, the methods used to decide which results to collect. | 2.3 Data extraction/synthesis |
|  | 10b | List and define all other variables for which data were sought (e.g. participant and intervention characteristics, funding sources). Describe any assumptions made about any missing or unclear information. | 2.3 Data extraction/synthesis |
| Study risk of bias assessment | 11 | Specify the methods used to assess risk of bias in the included studies, including details of the tool(s) used, how many reviewers assessed each study and whether they worked independently, and if applicable, details of automation tools used in the process. | 2.3 Data extraction/synthesis |
| Effect measures | 12 | Specify for each outcome the effect measure(s) (e.g. risk ratio, mean difference) used in the synthesis or presentation of results. | N/A |
| Synthesis methods | 13a | Describe the processes used to decide which studies were eligible for each synthesis (e.g. tabulating the study intervention characteristics and comparing against the planned groups for each synthesis (item #5)). | 2.2 Screening |
|  | 13b | Describe any methods required to prepare the data for presentation or synthesis, such as handling of missing summary statistics, or data conversions. | 2.3 Data extraction/synthesis |
|  | 13c | Describe any methods used to tabulate or visually display results of individual studies and syntheses. | 2.3 Data extraction/synthesis |
|  | 13d | Describe any methods used to synthesize results and provide a rationale for the choice(s). If meta-analysis was performed, describe the model(s), method(s) to identify the presence and extent of statistical heterogeneity, and software package(s) used. | 2.3 Data extraction/synthesis |
|  | 13e | Describe any methods used to explore possible causes of heterogeneity among study results (e.g. subgroup analysis, meta-regression). | N/A |
|  | 13f | Describe any sensitivity analyses conducted to assess robustness of the synthesized results. | N/A |
| Reporting bias assessment | 14 | Describe any methods used to assess risk of bias due to missing results in a synthesis (arising from reporting biases). | N/A |
| Certainty assessment | 15 | Describe any methods used to assess certainty (or confidence) in the body of evidence for an outcome. | N/A |
| **RESULTS** | | |  |
| Study selection | 16a | Describe the results of the search and selection process, from the number of records identified in the search to the number of studies included in the review, ideally using a flow diagram. | Figure 1 |
|  | 16b | Cite studies that might appear to meet the inclusion criteria, but which were excluded, and explain why they were excluded. | N/A |
| Study characteristics | 17 | Cite each included study and present its characteristics. | Table 2 |
| Risk of bias in studies | 18 | Present assessments of risk of bias for each included study. | Appendix C |
| Results of individual studies | 19 | For all outcomes, present, for each study: (a) summary statistics for each group (where appropriate) and (b) an effect estimate and its precision (e.g. confidence/credible interval), ideally using structured tables or plots. | N/A |
| Results of syntheses | 20a | For each synthesis, briefly summarise the characteristics and risk of bias among contributing studies. | Appendices C, D |
|  | 20b | Present results of all statistical syntheses conducted. If meta-analysis was done, present for each the summary estimate and its precision (e.g. confidence/credible interval) and measures of statistical heterogeneity. If comparing groups, describe the direction of the effect. | N/A |
|  | 20c | Present results of all investigations of possible causes of heterogeneity among study results. | N/A |
|  | 20d | Present results of all sensitivity analyses conducted to assess the robustness of the synthesized results. | N/A |
| Reporting biases | 21 | Present assessments of risk of bias due to missing results (arising from reporting biases) for each synthesis assessed. | N/A |
| Certainty of evidence | 22 | Present assessments of certainty (or confidence) in the body of evidence for each outcome assessed. | N/A |
| **DISCUSSION** | | |  |
| Discussion | 23a | Provide a general interpretation of the results in the context of other evidence. | Discussion |
|  | 23b | Discuss any limitations of the evidence included in the review. | 3.8 Quality assessment |
|  | 23c | Discuss any limitations of the review processes used. | 4.1 Limitations |
|  | 23d | Discuss implications of the results for practice, policy, and future research. | Discussion |
| **OTHER INFORMATION** | | |  |
| Registration and protocol | 24a | Provide registration information for the review, including register name and registration number, or state that the review was not registered. | Methods |
|  | 24b | Indicate where the review protocol can be accessed, or state that a protocol was not prepared. | Methods |
|  | 24c | Describe and explain any amendments to information provided at registration or in the protocol. | N/A |
| Support | 25 | Describe sources of financial or non-financial support for the review, and the role of the funders or sponsors in the review. | Funding |
| Competing interests | 26 | Declare any competing interests of review authors. | Conflicts of Interest |
| Availability of data, code and other materials | 27 | Report which of the following are publicly available and where they can be found: template data collection forms; data extracted from included studies; data used for all analyses; analytic code; any other materials used in the review. | Table 2 |

*From:*  Page MJ, McKenzie JE, Bossuyt PM, et al. The PRISMA 2020 statement: an updated guideline for reporting systematic reviews. BMJ 2021;372:n71. doi:10.1136/bmj.n71

For more information, visit: http://www.prisma-statement.org/

Appendix 2. Search Strategy

The search terms build upon the authors’ previous experience and existing literature reviews in the field. In addition, we consulted a health sciences librarian about our search strategy. To retrieve a full scope of the literature on our topic of interest, we imposed no time limit on years of publication. All three databases were searched on May 8, 2024.

PUBMED:

//Searched by titles and abstracts [tiab] and Medical Subject Headings [mh]

1 “dementia" [mh] OR dement* [tiab] OR Alzheimer* [tiab] OR ADRD [tiab] OR mild cognitive impairment* [tiab]

**2** "Visually Impaired Persons" [mh] OR "vision disorders" [mh] OR visually impaired [tiab] OR visual impairment* [tiab] OR vision disorder* [tiab] OR blindness [tiab] OR vision loss [tiab] OR visual loss [tiab] OR vision acuit* [tiab] OR visual acuit* [tiab] OR partial sight [tiab]

**3** "hearing loss" [mh] OR "persons with hearing impairments" [mh] OR hearing loss [tiab] OR hearing impairment* [tiab] OR hearing deficit* [tiab] OR hearing dysfunction* [tiab] OR hearing disorder* [tiab] OR hard of hearing [tiab] OR impaired hearing [tiab] OR loss of hearing [tiab] OR auditory impairment* [tiab] OR auditory deficit* [tiab] OR auditory dysfunction* [tiab] OR auditory acuit* [tiab] OR deaf* [tiab]

**4** "caregivers" [mh] OR caregiv* [tiab]

**5 2 OR 3**

6 1 AND 5 AND 4

CINAHL Plus with Full Text, and PsycINFO:

//Searched by titles and abstracts

1 **dement* OR Alzheimer* OR ADRD OR mild cognitive impairment***

**2 visually impaired OR visual impairment* OR vision disorder* OR blindness OR vision loss OR visual loss OR vision acuit* OR visual acuit* OR partial sight**

**3 hearing loss OR hearing impairment* OR hearing deficit* OR hearing dysfunction* OR hearing disorder* OR hard of hearing OR impaired hearing OR loss of hearing OR auditory impairment* OR auditory deficit* OR auditory dysfunction* OR auditory acuit* OR deaf***

**4 caregiv***

**5 2 OR 3**

6 1 AND 5 AND 4

Appendix 3. Quality Assessment of the 12 Papers in the Final Sample

Joanna Briggs Institute’s critical appraisal checklist for qualitative studies

| Study | Lawrence et al.^25^ | Bunn et al.^23^ | Nyman et al.^28^ | Wolski et al.^31^ |
| --- | --- | --- | --- | --- |
| 1. Is there congruity between the stated philosophical perspective and the research methodology? | 0 | 1 | 1 | 0 |
| 2. Is there congruity between the research methodology and the research question or objectives? | 1 | 1 | 1 | 1 |
| 3. Is there congruity between the research methodology and the methods used to collect data? | 1 | 1 | 1 | 1 |
| 4. Is there congruity between the research methodology and the representation and analysis of data? | 1 | 1 | 1 | 1 |
| 5. Is there congruity between the research methodology and the interpretation of results? | 1 | 1 | 1 | 1 |
| 6. Is there a statement locating the researcher culturally or theoretically? | 1 | 1 | 1 | 0 |
| 7. Is the influence of the researcher on the research, and vice-versa, addressed? | 0 | 1 | 0 | 1 |
| 8. Are participants, and their voices, adequately represented? | 1 | 1 | 1 | 1 |
| 9. Is the research ethical according to current criteria or, for recent studies, and is there evidence of ethical approval by an appropriate body? | 0 | 1 | 1 | 1 |
| 10. Do the conclusions drawn in the research report flow from the analysis, or interpterion, of the data? | 1 | 1 | 1 | 1 |
| Rating | 70% fair | 100% excellent | 90% good | 80% good |

Joanna Briggs Institute’s critical appraisal checklist for cross-sectional studies

| Study | Varadaraj et al.^30^ | Powell et al.^29^ |
| --- | --- | --- |
| 1. Were the criteria for inclusion in the sample clearly defined? | 1 | 1 |
| 2. Were the study subjects and the setting described in detail? | 1 | 1 |
| 3. Was the exposure measured in a valid and reliable way? | 1 | 1 |
| 4. Were objective, standard criteria used for measurement of the condition? | 1 | 1 |
| 5. Were confounding factors identified? | 1 | 1 |
| 6. Were strategies to deal with confounding factors stated? | 1 | 1 |
| 7. Were the outcomes measured in a valid and reliable way? | 1 | 1 |
| 8. Was appropriate statistical analysis used? | 1 | 1 |
| Rating | 100% excellent | 100% excellent |

Joanna Briggs Institute’s critical appraisal checklist for quasi-experimental studies

| Study | Mamo et al.^6^ | Leroi et al.^26^ |
| --- | --- | --- |
| 1. Is it clear in the study what is the “cause” and what is the “effect” (i.e. there is no confusion about which variable comes first)? | 1 | 1 |
| 2. Was there a control group? | 0 | 0 |
| 3. Were participants included in any comparisons similar? | 1 | 1 |
| 4. Were the participants included in any comparisons receiving similar treatment/care, other than the exposure or intervention of interest? | 1 | 1 |
| 5. Were there multiple measurements of the outcome, both pre and post the intervention/exposure? | 0 | 0 |
| 6. Were the outcomes of participants included in any comparisons measured in the same way? | 1 | 1 |
| 7. Were outcomes measured in a reliable way? | 1 | 1 |
| 8. Was follow-up complete and if not, were differences between groups in terms of their follow-up adequately described and analyzed? | 0 | 0 |
| 9. Was appropriate statistical analysis used? | 1 | 1 |
| Rating | 67% fair | 67% fair |

Joanna Briggs Institute’s critical appraisal checklist for randomized controlled trials

| Study | Adrait et al.^22^ | Jorgensen et al.^24^ |
| --- | --- | --- |
| 1. Was true randomization used for assignment of participants to treatment groups? | 1 | 1 |
| 2. Was allocation to treatment groups concealed? | 1 | 0 |
| 3. Were treatment groups similar at the baseline? | 1 | 0 |
| 4. Were participants blind to treatment assignment? | 1 | 0 |
| 5. Were those delivering the treatment blind to treatment assignment? | 0 | 0 |
| 6. Were treatment groups treated identically other than the intervention of interest? | 1 | 1 |
| 7. Were outcome assessors blind to treatment assignment? | 1 | 0 |
| 8. Were outcomes measured in the same way for treatment groups? | 1 | 1 |
| 9. Were outcomes measured in a reliable way | 1 | 1 |
| 10. Was follow up complete and if not, were differences between groups in terms of their follow up adequately described and analysed? | 0 | 1 |
| 11. Were participants analysed in the groups to which they were randomized? | 1 | 1 |
| 12. Was appropriate statistical analysis used? | 1 | 0 |
| 13. Was the trial design appropriate and any deviations from the standard RCT design (individual randomization, parallel groups) accounted for in the conduct and analysis of the trial? | 1 | 0 |
| Rating | 85% good | 46% poor |

Mixed Methods Appraisal Tool, version 2018

| Study | Meyer et al.^27^ | Leroi et al.^5^ |
| --- | --- | --- |
| S1. Are there clear research questions? | 1 | 1 |
| S2. Do the collected data allow to address the research questions? | 1 | 1 |
| 1.1. Is the qualitative approach appropriate to answer the research question? | 1 | 1 |
| 1.2. Are the qualitative data collection methods adequate to address the research question? | 1 | 1 |
| 1.3. Are the findings adequately derived from the data? | 1 | 1 |
| 1.4. Is the interpretation of results sufficiently substantiated by data? | 1 | 1 |
| 1.5. Is there coherence between qualitative data sources, collection, analysis, and interpretation? | 1 | 1 |
| 4.1. Is the sampling strategy relevant to address the research question? | 1 | 1 |
| 4.2. Is the sample representative of the target population? | 0 | 1 |
| 4.3. Are the measurements appropriate? | 1 | 1 |
| 4.4. Is the risk of nonresponse bias low? | 1 | 1 |
| 4.5. Is the statistical analysis appropriate to answer the research question? | 1 | 1 |
| 5.1. Is there an adequate rationale for using a mixed methods design to address the research question? | 1 | 1 |
| 5.2. Are the different components of the study effectively integrated to answer the research question? | 1 | 1 |
| 5.3. Are the outputs of the integration of qualitative and quantitative components adequately interpreted? | 1 | 1 |
| 5.4. Are the divergences and inconsistencies between quantitative and qualitative results adequately addressed? | 0 | 1 |
| 5.5. Do the different components of the study adhere to the quality criteria of each tradition of the methods involved? | 1 | 1 |
| Rating | 88% good | 100% excellent |
